# 

**Published:** 1890-11

**Authors:** 


Entered at the Post Office at Austin, Texas, as Second-Class Mail Matter.

Vol. VI. NOVEMBER, 1890. No. 3.

### DANIEL'S TEXAS MEDICAL JOURNAL

F. E. DANIEL, M. D., Editor and Publisher, AUSTIN, TEXAS.

Eugene Von Boeckmann, Steam Book and Job Printer, Austin, Texas.

